# Victimization of People With Severe Mental Illness Outside and Within the Mental Health Care System: Results on Prevalence and Risk Factors From a Multicenter Study

**DOI:** 10.3389/fpsyt.2020.563860

**Published:** 2020-09-08

**Authors:** Verena Rossa-Roccor, Peter Schmid, Tilman Steinert

**Affiliations:** ^1^ School of Population and Public Health, University of British Columbia, Vancouver, BC, Canada; ^2^ Department of Psychiatry and Psychotherapy I Weissenau, Ulm University, Ravensburg, Germany

**Keywords:** victimization in psychiatry, risk factors for violence in mental health care system, illegal offences against patients, violence in mental health care system, patients as victims

## Abstract

We performed a cross-sectional study using a self-reporting survey to assess lifetime violent and non-violent victimization in people with severe mental illness experienced both inside (i.e., any service providing mental health care such as psychiatric hospitals, psychosocial rehabilitative programs, or outpatient care) and outside (i.e., in the personal life of the participants) of the mental health care system. We recruited 170 participants from 20 community mental health facilities. We built logistic regression models to assess potential risk factors for victimization inside the mental health care system. Outside of the mental health care system, the most commonly reported events were theft (n=93, 54.7%), physical violence without use of a weapon (n=87, 51.2%), and sexual harassment (n=82, 50.6%). Within the mental health care system, most commonly reported incidents were theft (n=68, 40.0%), sexual assault (n=18, 10.6%), and physical violence (n=47, 27.7%) by other patients or staff. Significant risk factors for specific victimization events inside the mental health care system were psychotic disorder, victimization in childhood and youth, female gender, number of hospitalizations, and duration of illness. Findings call for increased attention to victimization of people with severe mental illness, especially within the mental health care system as such victimization events may severely impact patients’ trajectories.

## Introduction

People with a mental health disorder have a significantly higher risk of becoming victims of violence compared to the general population (1–6). Previous research has focused largely on violent crimes such as physical assault, aggravated acquisitive crimes, violent threats, and sexual offenses in this population. Fewer studies have observed rates of victimization of non-violent crimes such as theft, robberies, or threats in this population for which the elevated risk compared to the general population persists (2). Significant differences between male and female individuals have also been described in the general population and among people with severe mental illness: men are more often victims of violence overall while women are more likely to be victims of domestic violence and sexual offenses (3, 7).

Within the mental health care system (i.e., treatment or support facilities that clients have personal contact with, for example in-patient hospital units, outpatient treatment, psychosocial rehabilitation programs), data on victimization is scarce and the prevalence of crimes against patients in the mental health care setting remains largely unknown. One previous study of US American outpatient clients (n=142) found a high prevalence of reported lifetime victimization within the mental health care system, with 31% reporting physical assault, 8% reporting sexual assault, and 63% witnessing traumatic events (8). Exposure to institutional violence (i.e., coercive measures such as being taken down by police or psychiatric staff, being committed against own will, forced medication, seclusion, or restraint) within the mental health care system has been shown to lead to re-traumatization and aggravation of mental health symptoms especially when a staff contact person was absent or treatment by staff was dismissive and derogatory (9–11). This potentially leads to a vicious circle of increased symptom severity, poorer outcome, and vulnerability for victimization (9, 12). Arguably, exposure to violence within the mental health care system that constitutes a criminal offense may lead to similar outcomes. It is therefore important to uncover the degree to which such events take place.

Not much is known about risk factors for exposure to violence in mental health care settings. Rather, the focus of previous studies has been on identifying risk factors for patients to become perpetrators rather than victims of violence while in psychiatric care. Some literature suggests that mentally ill individuals actually have a much higher risk of becoming victims rather than perpetrators of violence (13, 14). For example, Choe et al. (15) reported numbers between 2 to 13% of violent offenses conducted by mentally ill *vs.* 20 to 34% of mentally ill who had been victims of violent crimes during the same period. Some studies suggest that perpetration is highly correlated with victimization in people with severe mental illness (14). However, more recent population-based research has raised the hypothesis that perpetration is more likely to be a mediator on the causal pathway between mental health disorder onset and victimization (e.g., through sharing similar third variable risk factors) rather than presenting a true confounder for the relationship (2).

Victimization events across the lifespan are highly correlated, particularly for sexual revictimization (16–19). We therefore hypothesized that increased experience of victimization outside of the mental health care system (i.e., events in the participants’ personal life that were unrelated to services received from the mental health care sector) may be associated with elevated prevalence of victimization within the system. Steinert et al. (20) have shown that, among individuals with schizophrenic disorders, those who reported a history of trauma were significantly more often subjected to seclusion and restraint than those who did not have a history of trauma. Frueh et al. (8) found that lifetime exposure to sexual assault in individuals with severe mental illness was associated with certain experiences within the mental health care system, such as having medication used as a threat or sexual assault by a staff member.

The purpose of this cross-sectional study was a detailed assessment of the prevalence of different types of violent and non-violent victimization outside and within the mental health care system within the same sample. Furthermore, we evaluated whether exposure to violence outside of the mental health care system that preceded the first inpatient hospitalization was associated with an elevated risk for victimization within the mental health care system, controlling for known risk factors.

## Methods

### Participants

The target population for this study was adults with severe mental illness, whereby severity was defined by the need for social welfare support due to a mental health disorder (according to Germany’s Social Security Code XII). Participants were recruited through convenience sampling from facilities of the outpatient mental health care system in Southern Germany which provide clients with specific services such as supported living or vocational services. This strategy allowed us to depart from a severity definition that strictly focuses on diagnosis and instead included participants that were severely impacted in the social and occupational functioning, independent of their psychiatric diagnosis.

### Study Design and Research Procedures

This study was a cross-sectional, multicenter study which included 20 community mental health care facilities from 10 counties in Southern Germany. These included facilities of inpatient and outpatient assisted living, facilities that provide employment opportunities in the secondary labor market, and other psychosocial rehabilitation services. Inclusion criteria for participants were: receipt of social integration or caregiving services according to Social Security Code XII, residence in 1 of the 10 counties included in the study, and voluntary participation. Exclusion criteria were: limited cognitive function (not capable to fill out the questionnaire) and minority (under 18 years of age). After having received approval by the ethics commission of Ulm University, data collection through paper-based questionnaires was done from July to December 2014.

### Questionnaire

The *Weissenau Questionnaire on Victimization of People with Severe Mental Illness* is an extensive self-report questionnaire developed for the purpose of this study. Two pre-existing instruments were translated and modified to fit regional circumstances. The previously validated *Trauma Assessment for Adults—Self Report Version* (TAA; (21)) was used for the survey of trauma and victimization in participants’ personal lives. The original 17 items were reduced to ten items by excluding questions about traumatizing military experiences due to a low prevalence in the German population as well as questions about the relationship between victim and perpetrator as this was beyond the scope of this study. For the survey of victimization within the mental health care system, the *Psychiatric Experiences Questionnaire* (PEQ; (8)) was used in a modified version which excluded questions about consensual relationships with other patients or staff. Both questionnaires were translated and retranslated by two individuals who are fluent in both English and German.

The final questionnaire consisted of 49 items (detailed list of items see **Supplementary Table 1**) of which 10 pertained to participant characteristics, 10 assessed victimization in participants’ personal lives, and 29 inquired about victimization within the mental health care system which included coercive measures and incidents that constitute a criminal offense. For each item, participants were asked to indicate whether or not they had ever experienced a certain event and if so, how often. They were further asked to report when the first and last time of this experience took place in order to infer the temporal relationship between exposure to the victimization event and first hospitalization.

### Statistical Analysis and Study Variables

For descriptive purposes, we reported continuous variables as mean and standard deviation (SD) for normally distributed variables and as median with inter-quartile range (IQR) for non-normally distributed variables. For categorical variables, we reported frequencies. We applied two sample t-test to compare numeric variables such as number of occurrences of victimization events between female and male participants. For variables that were not normally distributed, we applied the non-parametric Mann-Whitney-U test. We compared categorical variables using Pearson’s chi square test, or Fisher’s exact test if assumptions were not met. We stratified descriptive statistics by gender. Answers such as “I don’t know,” “prefer not to say,” or “I don’t remember” were counted as missing observations. All results are based on complete case analysis excluding missing observations per item.

We analyzed the association between victimization outside and victimization within the mental health care system through multiple logistic regression models for these main explanatory variables: crimes against property outside (model 1); physical violence outside (model 2); and sexual violence outside the mental health care system (model 3). Each of these models was calculated for five outcomes of victimization inside the mental health care system: a) crimes against property, b) lack of privacy and adequate nutrition, c) physical violence, d) sexual violence, e) coercive measures, resulting in model labels 1a-e, 2a-e, and 3a-e (see **Table 4**). The main explanatory and outcome variables included in the models were created as thematic aggregate scores of individual questionnaire items pertaining to these variables. For better interpretability, we additionally conducted a Poisson regression with a composite model aggregating a) to d) into one outcome variable of victimization within the mental health care system. The covariates and main explanatory variables remained the same. Based on our conceptual understanding, findings in previous literature, and exclusion of multicollinearity through Pearson’s correlation coefficient, we included the following as covariates in each of the models: age, gender, and living situation to reflect sociodemographic status; psychotic and substance use disorder as known risk factors for victimization compared to other diagnoses (2, 22); victimization in childhood and youth as known risk factor for victimization across the lifespan (1, 16–19); and finally, duration of illness and number of hospitalizations. We considered temporality between the experiences outside and those within the mental health care setting by only including observations of cases where at least one occurrence of victimization outside the mental health care system preceded the first hospitalization. Observations with no previous exposure to violence were fully included in the models as the reference category. Model fit was assessed through pseudo R^2^. All statistical analysis was done using Statistica^®^ (StatSoft, Version 8.0).

## Results

### Response Rates and Participant Characteristics

The average response rate among recruited participants across all facilities was 44.8%. The total study sample consisted of 170 participants of which 50% were women (n=85) and 50% were men (n=85). The average age of participants was 44.2 years (± 12.1 years) with an average duration of illness of 23.1 years (± 9.8). Psychosis (44.7%) and affective disorders (41.2%) represented the most prevalent disorders in the study sample. Substance use was reported significantly more often by male participants (27.1 *vs.* 10.6%, *p*=0.006). Participants were hospitalized a median number of 5.0 times (IQR 2.0–10.0) and the last hospitalization preceded the time of data collection by a median of 2.0 years (IQR=0.8–4.0). Further details are shown in **Table 1**.

**Table 1:** Sociodemographic and mental health disorder-related variables in a multicenter sample of 170 people with severe mental illness.

	Male (n=85)		Female (n=85)		Total (n=170)	
Item						
	(Mean ± SD)		(Mean ± SD)		(Mean ± SD)	
Age	44.3 ± 11.9		44.1 ± 12.4		44.2 ± 12.1	
	n	%	n	%	n	%
Diagnosis^1^						
*Psychosis*	41	48.2%	35	41.2%	76	44.7%
*Personality disorder*	25	29.4%	23	27.1%	48	28.2%
*Affective disorder*	30	35.3%	40	47.1	70	41.2%
*Substance use disorder*	23	27.1%	9	10.6%	32	18.8%
*Other*	9	10.6%	18	21.2%	27	15.9%
Housing^2^						
*Independently in own home*	26	30.6%	31	26.5%	57	33.5%
*With own family*	3	3.5%	12	14.11%	15	8.8%
*With birth family*	3	3.5%	1	1.2%	4	2.4%
*With host family^3^*	2	2.4%	0	0.0%	2	1.2%
*Outpatient assisted living*	26	30.6%	30	35.3%	56	32.9%
*Inpatient assisted living*	31	36.5%	18	21.2%	49	28.8%
*Other*	4	4.7%	3	3.5%	7	4.1%
Nationality						
*German*	78	91.8%	78	91.8%	156	91.8%
*Non-German*	7	8.2%	3	3.5%	10	5.9%
*Dual citizenship (German + other)*	0	0.0%	3	3.5%	3	1.8%
	(Mean ± SD)		(Mean ± SD)		(Mean ± SD)	
Age of onset	23.6 ± 9.1		21.3 ± 10.4		22.4 ± 9.8	
	(Median; IQR)		(Median; IQR)		(Median; IQR)	
Age at time of first hospitalization	25.0; 20.0–32.8		22.0; 18.0–32.3		25.0; 19.0–33.8	
Number of hospitalizations	5.0; 3.0–10.0		5.0; 2.0–10.0		5.0; 2.0–10.0	
Time (in years) since last hospitalization	2.0; 0.5–4.0		2.0; 1.0–4.0		2.0; 0.8–4.0	

^1^Multiple answers were allowed for this item in order to reflect co-morbidity.

^2^Multiple answers were allowed for this item, e.g., it is possible to live independently in one’s own apartment and receive outpatient assisted living support.

^3^Living with host families is a form of accommodation for mentally ill available in some regions of Germany. Patients live with families that are not their own who provide shelter, food, social support, basic care, and sometimes employment, e.g., in the form of farm work.

### Prevalence of Victimization Outside the Mental Health Care System for Total Sample and Compared by Gender

The results on prevalence of victimization events outside the mental health care system are presented in **Table 2**. Particularly high proportions (more than 50% of participants had experienced this at least once) were reported for theft (54.7%; n=93); threat of physical violence without a weapon (70.0%; n=119); physical violence without a weapon (51.2%; n=87); and sexual harassment (50.6%; n=82).

**Table 2:** Prevalence of victimization outside of the mental health care system in a multicenter sample of 170 people with severe mental illness.

Item	Male (n=85)		Female (n=85)		*p* male *vs.* female	df	Total sample (n=170)	
	n	%	n	%			n	%
Theft	47	55.3%	46	54.1%	0.88	1	93	54.7%
Burglary	15	17.6%	20	23.8%	0.32	1	35	20.6%
Threat of physical violence no weapon	59	69.4%	60	70.6%	0.04*	1	119	70.0%
Physical violence no weapon	38	44.7%	49	60.5%	0.04*	1	87	51.2%
Threat of violence with weapon	29	34.1%	33	38.8%	0.49	1	62	36.5%
Violence with weapon	8	9.5%	15	18.3%	0.10	1	23	13.5%
Violent situation with fear for life	31	36.5%	39	47.0%	0.17	1	70	41.2%
Witness violence on another person	32	37.6%	31	36.5%	0.78	1	63	37.1%
Sexual harassment	22	25.9%	60	70.6%	≤0.001*	1	82	50.6%
Rape	9	10.8%	43	51.2%	≤0.001*	1	52	30.6%

* = significant with a significance level of p ≤ 0.05.

A significantly higher proportion of women reported to have experienced sexual harassment (70.6%, n=60 *vs.* 25.9%, n=22 of men; *p ≤* 0.001), rape (51.2%, n=43 *vs.* 10.8%, n=9 of men; *p ≤* 0.001), and physical violence without a weapon (60.5%, n=49 *vs.* 44.7%, n=38 of men; *p*=0.04).

### Prevalence of Victimization in the Mental Health Care System for Total Sample and Compared by Gender

Detailed results on prevalence of victimization within the mental health care system are reported in **Table 3**. Three items had a prevalence of over 50% in the total sample: being around violent or frightening patients (51.2%, n=87), badgering or name calling by another patient (50.0%, n=85), and being committed against own will (55.9%, n=95). Incidents which constitute a criminal offense within the mental health care system were reported as follows: 40.0% (n=68) reported to have experienced theft; 7.1% (n=12) and 3.5% (n=6) reported having been sexually assaulted by another patient or staff, respectively; 17.1% (n=29) reported to have been physically assaulted by another patient; and 10.6% (n=18) reported to have experienced physical violence by staff (other than coercive measures or having been “taken down”).

**Table 3:** Prevalence of victimization within the mental health care system in a multicenter sample of 170 people with severe mental illness.

Item	Male (n=85)		Female (n=85)		*p* male *vs.* female	df	Total sample (n=170)	
	n	%	n	%			n	%
Lack of proper food/nutrition	9	10.7%	4	4.8%	0.16	1	13	7.6%
No adequate privacy	10	11.8%	13	15.3%	0.50	1	23	13.5%
Theft	34	40.0%	34	40.0%	0.95	1	68	40.0%
Being around violent or frightening patients	41	48.2%	46	54.1%	0.39	1	87	51.2%
Badgering, calling names, bullying by staff	20	23.5%	21	24.7%	0.86	1	41	24.1%
Witnessing another patient being badgered, called names, bullied by staff	14	16.5%	17	20.0%	0.58	1	31	18.2%
Badgering, calling names, bullying by another patient	46	56.1%	39	46.3%	0.21	1	85	50.0%
Taken down by police	30	36.6%	21	25.0%	0.11	1	51	30.0%
Witnessing other patients being taken down by police	22	25.9%	18	21.2%	0.44	1	40	23.5%
Taken down by staff	22	27.2%	28	33.3%	0.39	1	50	29.4%
Witnessing another patient being taken down by staff	42	49.2%	41	48.2%	0.82	1	83	48.8%
Threat of physical violence	36	43.9%	21	25.3%	0.01*	1	57	33.5%
Physical violence by staff	8	10.0%	10	11.9%	0.69	1	18	10.6%
Physical violence by another patient	14	16.5%	15	17.6%	0.89	1	29	17.1%
Witnessing physical violence by staff	13	15.3%	13	15.3%	0.92	1	26	15.3%
Witnessing physical violence by another patient	14	16.5%	16	18.8%	0.74	1	30	17.6%
Witnessing physical violence against staff	12	14.6%	17	20.2%	0.34	1	29	17.1%
Intrusive and unwanted sexual advances	9	10.8%	27	32.1%	≤0.001*	1	36	21.2%
Sexual assault by staff	1	1.2%	5	6.0%	0.09	1	6	3.5%
Sexual assault by another patient	3	3.7%	9	10.8%	0.07	1	12	7.1%
Witnessing of sexual assault by staff	0	0.0%	3	3.6%	0.25	1	3	1.8%
Witnessing sexual assault by another patient	2	2.4%	3	3.6%	0.67	1	5	2.9%
Committed against will	47	55.3%	48	65.5%	0.91	1	95	55.9%
Placed in seclusion against will	27	31.8%	28	32.9%	0.95	1	55	32.4%
Put in restraints	30	35.3%	34	40.0%	0.61	1	64	37.6%
Strip-searched	28	34.6%	16	19.8%	0.03*	1	44	25.9%
Given medication against will	20	24.4%	30	35.7%	0.11	1	50	29.4%
Threat of medication against will as punishment	11	12.9%	15	17.6%	0.41	1	26	15.3%
Witnessing of death of another person	21	24.7%	20	23.5%	0.82	1	41	24.1%

* = significant with a significance level of p ≤ 0.05.

Intrusive and unwanted sexual advances were reported by significantly more women (32.1%; n=27) than men (10.8%; n=9; *p ≤* 0.001). Men reported significantly higher proportions for threat of physical violence with 43.9% (n=36) *vs.* 25.3% of women (n=21, *p*=0.01) and strip-searched with 34.6% (n=28) of men and 19.8% (n=16, *p*=0.03) of women.

### Risk Factors for Victimization Within the Mental Health Care System

In the unadjusted models, the following incidents of criminal victimization outside were significantly associated with subsequent victimization within the mental health care system. Crimes against personal property were associated with a lack of adequate privacy and nutrition in the mental health care system (OR=2.67, 95% CI=1.15, 6.19). Sexual violence outside was associated with sexual violence within the mental health care system (OR=2.24, 95% CI=1.07, 4.70) as well as with a lack of adequate privacy and nutrition (OR=2.33, 95% CI=1.05, 5.17). When adjusting for all covariates (age, gender, living situation, psychotic disorder, substance use, victimization in childhood and youth, duration of illness, and number of hospitalizations), the associations between victimizations outside and those within the mental health care system did not remain statistically significant. Significant associations with the outcomes of different victimization events in the mental health care system were observed for several covariates. Detailed results are depicted in **Table 4** (to increase readability, we omitted covariates that were not significantly associated with any of the outcomes but were controlled for in the full models). To highlight a few significant findings: women have higher odds to become victims of sexual violence in the mental health care system compared to men (OR=3.09, 95% CI=1.78, 8.11 in model 1 and OR=2.80, 95% CI=1.09, 7.18 in model 2, respectively), while female gender was negatively associated with coercive measures (OR=0.19, 95% CI=0.05, 0.68 in model 1 and OR=0.18, 95% CI=0.05, 0.67 in model 2). We also found that the presence of a psychotic disorder increased odds of lack of privacy and adequate nutrition (OR=3.05, 95% CI=1.09, 8.47) and coercive measures (OR=3.59, 95% CI=1.03, 12.58) in model 1 and model 2 (lack of privacy and adequate nutrition: OR=2.76, 95% CI=1.01, 7.54 and coercive measures: OR=3.55, 95% CI=1.01, 12.47). In the composite model, we aggregated crimes against property, lack of proper nutrition and privacy, physical violence, and sexual violence within the mental health care system into one outcome variable. Here, we found that psychosis remained a statistically significant risk factor in model 1 and 3 (RR=1.37; 95% CI=1.02, 1.86 and RR=1.36; 95% CI=1.01, 1.84, respectively), while the number of hospitalizations was significantly associated with victimization within the mental health care system in model 2 (RR=1.01; 95% CI=1.00, 1.01).

**Table 4:** Adjusted and unadjusted logistic regression models of risk factors for victimization within the mental health care system in a multicenter sample of 170 people with severe mental illness.

	Outcomes (within the mental health care system)									
	a) Crimes against property^1^		b) Lack of privacy and adequate nutrition^1^		c) Physical violence^1^		d) Sexual violence^1^		e) Coercive measures^1^	
	Unadjusted OR (95% CI)	Adjusted OR (95% CI)	Unadjusted OR (95% CI)	Adjusted OR (95% CI)	Unadjusted OR (95% CI)	Adjusted OR (95% CI)	Unadjusted OR (95% CI)	Adjusted OR (95% CI)	Unadjusted OR (95% CI)	Adjusted OR (95% CI)
Model 1 (crimes against property outside of the mental health care system)*										
Crimes against property^2^ (reference: no)	2.09 (1.01, 4.30)	1.69 (0.70, 4.10)	**2.67 (1.15, 6.19)**	2.20 (0.76, 6.39)	1.98 (0.86, 4.51)	2.86 (0.97, 8.33)	1.47 (0.65, 3.33)	1.73 (0.64, 4.67)	1.05 (0.46, 2.38)	1.30 (0.35, 4.81)
Age (continuous)		0.96 (0.91, 1.00)		0.95 (0.89, 1.00)		1.01 (0.96, 1.06)		1.00 (0.95, 1.05)		**0.87 (0.80, 0.94)**
Gender (reference: male)		0.87 (0.39, 1.97)		1.27 (0.46, 3.53)		1.24 (0.47, 3.26)		**3.09 (1.78, 8.11)**		**0.19 (0.05, 0.68)**
Psychotic disorder (reference: no)		1.63 (0.73, 3.66)		**3.05 (1.09, 8.47)**		2.15 (0.84, 5.52)		1.48 (0.59, 3.72)		**3.59 (1.03, 12.58)**
Victimization in childhood and youth (reference: no)		1.49 (0.58, 3.89)		3.02 (0.71, 12.8)		0.78 (0.25, 2.41)		1.38 (0.45, 4.26)		**0.11 (0.02, 0.71)**
Duration of illness (continuous)		1.03 (0.99, 1.08)		1.05 (0.99, 1.11)		0.98 (0.94, 1.03)		0.99 (0.95, 1.04)		**1.22 (1.11, 1.34)**
Model 2 (physical violence outside of the mental health care system)**										
Physical violence^2^ (reference: no)	1.37 (0.74, 2.55)	0.74 (0.27, 2.00)	1.23 (0.56, 2.71)	0.41 (0.12, 1.39)	1.22 (0.64, 2.32)	0.85 (0.27, 2.67)	1.20 (0.58, 2.48)	0.66 (0.21, 2.05)	1.00 (0.50, 1.99)	1.40 (0.30, 6.48)
Age (continuous)		0.96 (0.91, 1.00)		0.95 (0.89, 1.01)		1.01 (0.96, 1.06)		1.00 (0.95, 1.05)		**0.87 (0.80, 0.94)**
Gender (reference: male)		0.80 (0.36, 1.78)		1.11 (0.40, 3.04)		0.96 (0.38, 2.39)		**2.80 (1.09, 7.18)**		**0.18 (0.05, 0.67)**
Psychotic disorder (reference: no)		1.54 (0.69, 3.44)		**2.76 (1.01, 7.54)**		1.92 (0.77, 4.80)		1.38 (0.56, 3.43)		**3.55 (1.01, 12.47)**
Victimization in childhood and youth (reference: no)		1.92 (0.68, 5.44)		**5.56 (1.12, 27.68)**		1.12 (0.36, 3.50)		1.87 (0.55, 6.36)		**0.11 (0.02, 0.71)**
Duration of illness (continuous)		1.02 (0.99, 1.05)		1.04 (0.97, 1.10)		0.98 (0.94, 1.03)		0.98 (0.94, 1.03)		**1.22 (1.11, 1.34)**
Number of hospitalizations (continuous)		1.02 (0.99, 1.05)		1.03 (0.99, 1.06)		**1.13 (1.03, 1.25)**		1.02 (0.99, 1.04)		Not measured^3^
Model 3 (sexual violence outside of the mental health care system)***										
Sexual violence^2^ (reference: no)	1.33 (0.70, 2.55)	1.19 (0.46, 3.08)	**2.33 (1.05, 5.17)**	2.36 (0.71, 7.81)	0.96 (0.49, 1.89)	0.79 (0.27, 2.32)	**2.24 (1.07, 4.70)**	1.41 (0.48, 4.16)	0.54 (0.27, 1.11)	0.62 (0.15, 2.54)
Age (continuous)		0.95 (0.91, 1.00)		**0.93 (0.87, 0.99)**		1.01 (0.96, 1.06)		0.99 (0.95, 1.05)		**0.88 (0.81, 0.95)**
Psychotic disorder (reference: no)		1.60 (0.71, 3.62)		**3.23 (1.14, 9.15)**		1.86 (0.74, 4.69)		1.48 (0.59, 3.74)		2.63 (0.72, 9.59)
Duration of illness (continuous)		1.03 (0.99, 1.08)		1.05 (0.99, 1.12)		0.98 (0.94, 1.03)		0.99 (0.95, 1.04)		**1.17 (1.07, 1.28)**
Number of hospitalizations (continuous)		1.02 (0.99, 1.05)		1.02 (0.99, 1.06)		**1.13 (1.03, 1.25)**		1.02 (0.99, 1.04)		Not measured^3^

^1^Inside the mental health care system.

^2^Outside of the mental health care system.

^3^Not measured due to too small of an n in at least one of the fields of the contingency table.

*R^2^_Model 1a_=0.08; R^2^_Model 1b_=0.16; R^2^_Model 1c_=0.17; R^2^_Model 1d_=0.09; R^2^_Model 1e_=0.40.

**R^2^_Model 2a_=0.08; R^2^_Model 2b_=0.16; R^2^_Model 2c_=0.15; R^2^_Model 2d_=0.08; R^2^_Model 2e_=0.40.

***R^2^_Model 3a_=0.08; R^2^_Model 2b_=0.16; R^2^_Model 3c_=0.15; R^2^_Model 3d_=0.08; R^2^_Model 3e_=0.43.

R^2^ values as a measure of model fit ranged between 8 and 43%. The highest R^2^ value was observed for the full model with coercive measures as outcome and sexual violence as main explanatory variable.

## Discussion

### Interpretation

This study shows a high prevalence of victimization among people with severe mental illness. The results confirm previous findings on victimization of people with mental illness (2–5) while they contribute a more detailed picture by combining violent and non-violent victimization outside and within the mental health care system within the same study population. To the best of our knowledge, there is only one previous study that assessed criminal offenses against patients within the mental health care setting. The authors reported incidents such as physical or sexual assault by another patient or staff and highlighted that although these events were reported by less than 10% of the participants, they were indeed highly distressing and have the potential to extremely impact the individual’s further trajectory, trust in the mental health care system, and quality of life (8). Although the prevalence of these experiences in our sample was similarly low, the mere occurrence of criminal offenses conducted by other patients and particularly by staff in psychiatric care—an environment where individuals are highly vulnerable and rightfully expect to be in a safe environment—give reason to be alert.

We hypothesized that exposure to victimization outside may be associated with increased reports of victimization within the mental health care system. While this was true for the unadjusted association in this study, none of the relationship between the exposures outside the mental health care system and those within remained significant once we controlled for covariates. Several of these covariates were significantly associated with victimization within the mental health care system in the full models and confirm previous studies. Female gender was strongly associated with sexual violence. This association has been described extensively in the literature on victimization of people with mental health disorders (3, 7, 23) and can also be seen in the general population, although women with severe mental illness are more likely to become the victim of sexual violence than women in the general population (7). Psychotic disorder as a risk factor for coercive measures is a well-known phenomenon (20, 24), although its association with other victimization events within the mental health care system has not previously been assessed. This may add to the conceptualization of this particular group being highly vulnerable for victimization, even within the mental health care system. However, it is also possible that this may be reported more often among this population due to underlying symptoms such as paranoia. We observed high odds for those who experience childhood trauma for lack of privacy and adequate nutrition in only one model (physical violence), although this observation must be interpreted with caution due to a large 95% CI. Importantly, a perceived lack of adequate privacy and nutrition may vary widely not only due to the symptoms of the patients but also due the type of facility (e.g., outpatient *vs.* inpatient) and treatment received. For example, the standard treatment of substance use often involves intensive monitoring and can be perceived as a severe intrusion of privacy—potentially meaning that clinicians may need to more carefully consider whether these measures are justified. Many mental health care facilities also operate at or beyond capacity which may also contribute to a lack of privacy. Finally, duration of illness and number of hospitalizations were significantly associated with coercive measures and physical violence, respectively. Both are likely connected to victimization through unmeasured factors such as symptom severity and chronicity (6). It is worth noting that in our study sample living situation did not seem to play a significant role in predicting victimization in the mental health care system. This seems contrary to an extensive body of literature that describes housing as an important risk factor for victimization (25, 26). However, our study sample was recruited from individuals who live in Germany where people tend to receive more continuous mental health care and social security services. None of our participants were therefore vulnerably housed and 61.8% lived in some form of assisted living arrangement. In addition, although the data collected in this study focuses on patient-related characteristics only, however, this is not to suggest that victimization occurs solely based on factors related to the patient but is likely dependent on external factors such as the type of facility, disorders present among other patients, and factors that influence staff behavior such as workload or de-escalation training. Finally, it is worth pointing out that, despite the high prevalence of victimization events reported in this sample, merely n=6 (3.5%) of participants reported to suffer from or have been diagnosed with posttraumatic stress disorder (PTSD). This may be due to a negligence of exploring PTSD in people with severe mental illness in clinical practice. Given that a large proportion of patients has likely suffered from some, if not severe, forms of victimization, an increased attention to exploring PTSD as a possible co-morbidity in this patient population may be indicated.

### Strengths and Limitations

Limitations of this study are inherent to the cross-sectional study design which does not allow for conclusions about causal pathways. We made an effort to partially attenuate this concern by introducing a temporal sequence—the analysis included only observations of exposure to violence outside the mental health system when at least one event preceded the first hospitalization. The relatively poor model fit of some of the models in this study indicates a more complex relationship between potential risk factors and victimization with the mental health care system and the presence of unmeasured factors. Both the relatively small sample size (n=170) and selection bias may present a limitation to the generalizability of the results. For example, it is possible that only individuals who had suffered a disproportionate amount of victimization chose to participate in the study in order to be able to share their experiences. As a non-random sampling strategy was used, there is likely some variability in the sociodemographic variables between the study sample and the general population of people with severe mental illness. However, this sampling strategy was chosen in order to obtain a study sample that is representative in terms of their illness-related functioning. Participants were recruited from facilities which service patients and clients who receive benefits according to Germany’s social security code XII which, per definition, is only granted to individuals with severe limitations of their functionality. This approach made it possible to recruit a group of severely disabled participants who lived in a wide range of different living conditions in the care of 20 different facilities in ten counties and allowed us to depart from the narrow diagnosis-related definition of severe mental illness, which is generally defined as having schizophrenia, bipolar disorder, or major depression. The use of self-reported data limits the reliability of the results and further introduces an information bias. Details about quantity and time of certain experiences are subject to recall bias and can certainly only represent individual estimates. Indeed, we observed a high proportion of missing data and a wide variation in answers with regards to the frequency of occurrences of certain events. This compelled us to conceptualize the outcome variables as dichotomous (i.e., yes/no) rather than count variables which would have rendered a more detailed picture of the individual victimization events. In addition, certain psychopathologies such as delusion can further skew an individual’s answers to the survey questions. However, we believe that the wide heterogeneity of participants and the considerable average timely distance to the last hospitalization with acute symptoms minimizes this bias. Further limitations to the study were caused by the use of a survey that was not previously tested for validity or reliability; however, the survey was meant to primarily assess prevalence rather than measure psychological factors.

### Conclusion and Future Research

People with severe mental illness are vulnerable to becoming victims of violent and non-violent crimes. Our findings reflect that this is also true within the mental health care system. This is concerning since patients rightfully expect a safe environment when receiving care. We further show that certain groups (women, people with psychotic disorders, patients with a long history of mental illness) may be particularly at risk for victimization in the mental health care setting. Evidence shows that experiencing victimization contributes to a destabilization, prolongation, and exacerbation of the disease. However, in our experience, there is little focus on traumatic events of patients in routine psychiatric care and histories of victimization, especially those that occurred within psychiatric care, are often being overlooked. Practitioners may thus consider introducing questions about prior experience of violence in routine anamnesis which may help uncover comorbidities with PTSD and improve the delivery of trauma-informed care.

The results of this study are preliminary. Further research is certainly indicated to paint a more complete picture of risk factors contributing to victimization experiences, especially with respect to the occurrence of criminal offenses within the mental health care system. Given that many people with severe mental illness are often and for long periods of time dependent on psychiatric care, this is an important component of trauma history. Although we based our models on existing literature and a conceptual understanding, the model fit of some of the herein presented results is relatively poor pointing at more complex underlying pathways. More factors such as socioeconomic status or vulnerable housing may be taken into consideration. Furthermore, it is important to investigate non-patient related factors such as types of facilities (e.g., acute care *vs.* outpatient care) or perpetrator-related characteristics to ensure that victimization is not considered solely based on the patients’ characteristics. It would be informative to explore mediating pathways between lifetime exposure to victimization and victimization that happens specifically within mental health care facilities. Finally, future research may focus on protective factors to inform clinicians about approaches to preventing victimization and traumatization of patients.

## Data Availability Statement

The datasets presented in this article are not readily available because data sharing was not included in the written consent of participants. Requests to access the datasets should be directed to verena.rossa-roccor@ubc.ca.

## Ethics Statement

The studies involving human participants were reviewed and approved by Ethics commission of Ulm University (Ethikkommission der Universitaet Ulm). The participants provided their written informed consent to participate in this study.

## Author Contributions

VR-R: Conceptualization, methodology, validation, support in formal analysis, investigation, writing —original draft. PS: methodology, validation, formal analysis lead, writing—review and editing. TS: Conceptualization, methodology, validation, writing—review and editing, supervision.

## Conflict of Interest

The authors declare that the research was conducted in the absence of any commercial or financial relationships that could be construed as a potential conflict of interest.
