# Supplementary material for: Victimization of People With Severe Mental Illness Outside and Within the Mental Health Care System: Results on Prevalence and Risk Factors From a Multicenter Study
Source: Front Psychiatry. 2020 Sep 8;11:563860. doi: 10.3389/fpsyt.2020.563860 (PMC7509533; doi:10.3389/fpsyt.2020.563860)
Supplement: Supplementary file 1 [file Table_1.docx]

Supplementary Material

# Supplementary Table

Supplementary Table 1. List of items in the Weissenau Questionnaire on Victimization of People with Severe Mental Illness

| Item # | Item text (English translation) |
| --- | --- |
| *Section I – Sociodemographic variables and information pertaining to trajectory of psychiatric illness* | |
| I.1. | Age |
| I.2. | Gender |
| I.3. | Nationality |
| I.4. | Reason for psychiatric treatment (more than one answer possible)? |
| I.5. | How old were you when your symptoms started? |
| I.6. | How often have you been hospitalized because of your psychiatric symptoms? |
| I.7. | How old were you when you were first hospitalized because of your psychiatric symptoms? |
| I.8. | When (what year) was your last hospitalization because of your psychiatric symptoms? |
| I.9. | What is your living situation? |
| I.10. | In general, how well do you follow specific psychiatric recommendations  (e.g., medications, therapy, etc.)? |
| *Section II – Victimization events outside of the mental health care system* | |
| II.1. | Has anyone ever stolen or tried to steal your personal belongings (such as wallet, cell phone etc.)? |
| II.2. | Has anyone ever broken into your home or place of residence? |
| II.3. | Have you ever been threatened with physical violence without a weapon? |
| II.4. | Have you ever been seriously injured or suffered physical damage due to physical violence without a weapon? |
| II.5. | Have you ever been threatened with physical violence with a weapon (e.g., gun, knife, bat, etc.)? |
| II.6. | Have you ever been seriously injured or suffered physical damage due to physical violence with a weapon? |
| II.7. | Have you ever been in a situation of violence where you feared for your life? |
| II.8. | Have you ever witnessed murder or physical violence towards another person? |
| II.9. | Have you ever experienced sexual harassment? |
| II.10. | Have you ever been raped? |
| *Section III – Victimization events within the mental health care system* | |
| III.1. | Have you ever been deprived of adequate food or nutrition? |
| III.2. | Have you ever not had adequate privacy for bathing, dressing, or using the toilet? |
| III.3. | Have you ever had your personal belongings (e.g., wallet, cell phone, etc.) stolen or taken away? |
| III.4. | Have you ever been around other patients who were very violent of frightening in other ways? |
| III.5. | Have you ever experienced staff calling you names (e.g., ‘crazy’, ‘stupid’), badgering or bullying you in some other verbal way? |
| III.6. | Have you ever heard staff calling other patients names, badgering or bullying them in some other verbal way? |
| III.7. | Have you ever experienced other patients calling you names, badgering or bullying you in some other verbal way? |
| III.8. | Have you ever been ‘taken down’ by police? |
| III.9. | Have you ever witnessed another patient being ‘taken down’ by police? |
| III.10. | Have you ever been ‘taken down’ by psychiatric staff? |
| III.11. | Have you ever witnessed another patient being ‘taken down’ by psychiatric staff? |
| III.12. | Have you ever been threatened with physical violence? |
| III.13. | Have you ever experienced a physical assault (e.g. being hit, punched, slapped, kicked, strangled, burned, etc.) by a staff member? |
| III.14. | Have you ever experienced a physical assault (e.g. being hit, punched, slapped, kicked, strangled, burned, etc.) by another patient? |
| III.15. | Have you ever witnessed a physical assault on another patient by a staff member? |
| III.16. | Have you ever witnessed a physical assault on another patient by another patient? |
| III.17. | Have you ever witnessed a physical assault on a staff member by another patient? |
| III.18. | Have you ever experienced intrusive and unwanted sexual advances (e.g., someone talking to you about having sex, touching your body)? |
| III.19. | Have you ever experienced a sexual assault (e.g., pressure, threats, or force to engage in any type of sexual contact) by a staff member? |
| III.20. | Have you ever experienced a sexual assault (e.g., pressure, threats, or force to engage in any type of sexual contact) by another patient? |
| III.21. | Have you ever witnessed another patient being sexually assaulted (e.g., pressure, threats, or force to engage in any type of sexual contact) by a staff member? |
| III.22. | Have you ever witnessed another patient being sexually assaulted (e.g., pressure, threats, or force to engage in any type of sexual contact) by another patient? |
| III.23. | Have you ever been committed against your will or have you been threatened with commitment? |
| III.24. | Have you ever been placed in seclusion against your will? |
| III.25. | Have you ever been put in restraints of any kind against your will? |
| III.26. | Have you ever been strip-searched against your will? |
| III.27. | Have you ever been forced to take medication against your will? |
| III.28. | Have you ever had medication used as a threat or as punishment? |
| III.29. | Have you ever witnessed the death of another person? |
